# The complete mitochondrial genome of *Cochylimorpha cultana* (Lederer, 1855) (Lepidoptera: Tortricidae)

**DOI:** 10.1080/23802359.2021.1959432

**Published:** 2021-07-27

**Authors:** Zhoujie Qi, Yinghui Sun, Yongling Sun, Liyan Wang, Tisen Xu

**Affiliations:** College of Life Sciences, Dezhou University, Shandong, China

**Keywords:** *Cochylimorpha cultana*, mitochondrial genome, Tortricidae, phylogenetic analysis

## Abstract

The complete mitochondrial genome of *Cochylimorpha cultana* (Lederer) (Lepidoptera: Tortricidae) was 15,348 bps in size, and comprised 37 genes, which were 13 PCGs, 22 tRNA genes and two rRNA genes. Most PCGs used the conventional ATN start codon, except for *cox1* initiating with CGA. Four genes (*cox1*, *cox2*, *nad4* and *nad5*) used single T residue as stop condon. 21 out of 22 tRNAs are folded into the cloverleaf secondary structure, except for *trnS1*. The phylogenetic analysis based on maximum-likelihood (ML) method revealed that the evolutionary status of *C. cultana* in Tortricinae at the molecular level, which agrees well with the classical taxonomy.

The leaf roller moth *Cochylimorpha cultana* belongs to the genus *Cochylimorpha* Razowski, 1959, which is one of the largest genera in the tribe Cochylini of subfamily Tortricinae. A recent phylogenetic analysis of the tribe placed *Cochylimorpha* as sister to *Eugnosta* Hübner, [1825] 1816 (Brown et al. 2019). At present, *Cochylimorpha* comprises 97 species worldwide (Gilligan et al. 2018), with greatest species richness in China, Russia and Europe (Sun and Li 2013). The larvae of *Cochylimorpha* utilize mainly Artemisia species (Asteraceae), often feeding on the seeds, stems, and roots (Razowski 1987). Numerous species are bound in open, dry biotopes, e.g. sands and various xerotherms; many species are found in the steppes (Razowski 2009).

Since the complete mitochondrial genome of *Adoxophyes honmai* was reported in 2006, there are currently 19 complete mitochondrial genomes of Tortricidae have been published (Lee et al. 2006; Son and Kim 2011; Zhao et al. 2011; Zhu et al. 2012; Shi et al. 2013; Wu et al. 2013; Niu et al. 2016; Piper et al. 2016; Wu et al. 2016; Zhao et al. 2016; Fagua et al. 2018; Ding et al. 2020; Xiang 2020). Herein, we sequenced mitochondrial genome of *C. cultana*, which provided sufficient basis for further analysis of phylogenetic and evolutionary relationship of Tortricidae.

In this study, adult individual of *C. cultana* was collected from Yanchi County, Ningxia Autonomous Region, China (37.93°N, 107.40°E) in 2017 by light trap. The specimen was identified according to Razowski (1970) and Sun and Li (2013). When the specimen was collected in the field, the three right legs were directly preserved in 95% ethanol and then stored at −20 °C. The remainders of the specimen were deposited as vouchers in the Insect Collection, College of Life Sciences, Dezhou University, Shandong, China (Yinghui Sun, sunyinghui8789@126.com), under the accession no. DZU001.

Genomic DNA was extracted from leg muscle using Rapid Animal Genomic DNA Isolation Kit (Sangon Biotech Co., Ltd., Shanghai, China). The genomic library is established and then used Illumina NovaSeq 6000 platform for high-throughput sequencing performed in Tianjin Novogene Technology Co., Ltd., China. The mitogenome assembly was carried out with the software SPAdes V.3.14.1 (Bankevich et al. 2012) and MitoZ V.2.3 (Meng et al. 2019). Sequence polish and correctness check were executed with Pilon V.1.23 (Walker et al. 2014). MITOS Web Server (http://mitos2.bioinf.uni-leipzig.de/index.py) was utilized for annotation of the mitogenome (Bernt et al. 2013; Cameron 2014). Geneious Prime 2020.2.2 was used to compare the homologous gene annotations of other insects and then submitted to NCBI (Kearse et al. 2012).

In this study, the circular mitogenome of *C. cultana* (GenBank accession number: MW413306) was sequenced, assembled and annotated. It was 15,348 bps in size, comprising 37 genes (13 protein-coding genes, 22 tRNA genes, two rRNA genes), in the same gene order as most Ditrysian moth mitogenomes (Park et al. 2016; Wang et al. 2018; Chen et al. 2020). The base composition of *C. cultana* mitogenome was strongly AT biased (AT 80.8%, CG 19.2%). Most PCGs of *C. cultana* were using ATN as start codon, while the only exception happened in *cox1* which started with putative CGA. With respect to PCG stop codon, the conventional TAA or TAG were most used, except for *cox1*, *cox2*, *nad4* and *nad5* genes which end with a single T residue, where the stop codon is completed by the addition of a poly-A tail to the mRNA. 21 out of 22 tRNAs exhibited the classic cloverleaf structure. *TrnS1* as the lone exception possessed a large loop where normally the DHU arm should have formed instead, and additionally, the anticodon of *trnS1* was ACU rather than the commonly used GCU. The 372 bp long control region was highly rich in AT content (96.5%).

A data set was built based on 13 PCGs of *C. cultana* plus 24 GenBank sequences from 15 genera, then it was imported into IQ-TREE software V.2.07 (Nguyen et al. 2015) to construct a ML tree (Figure 1), according to the optimal substitution models under the rapid bootstrap algorithm (1000 replicates). The tree generated showed that the monophyly of the two major subfamilies Olethreutinae and Tortricinae was well retained. This study verifies the evolutionary status of *C. cultana* in Tortricidae at the molecular level, which agreed with the classical taxonomy. The mitochondrial genome would be a significant supplement for the *C. cultana* genetic background.

**Figure 1.**
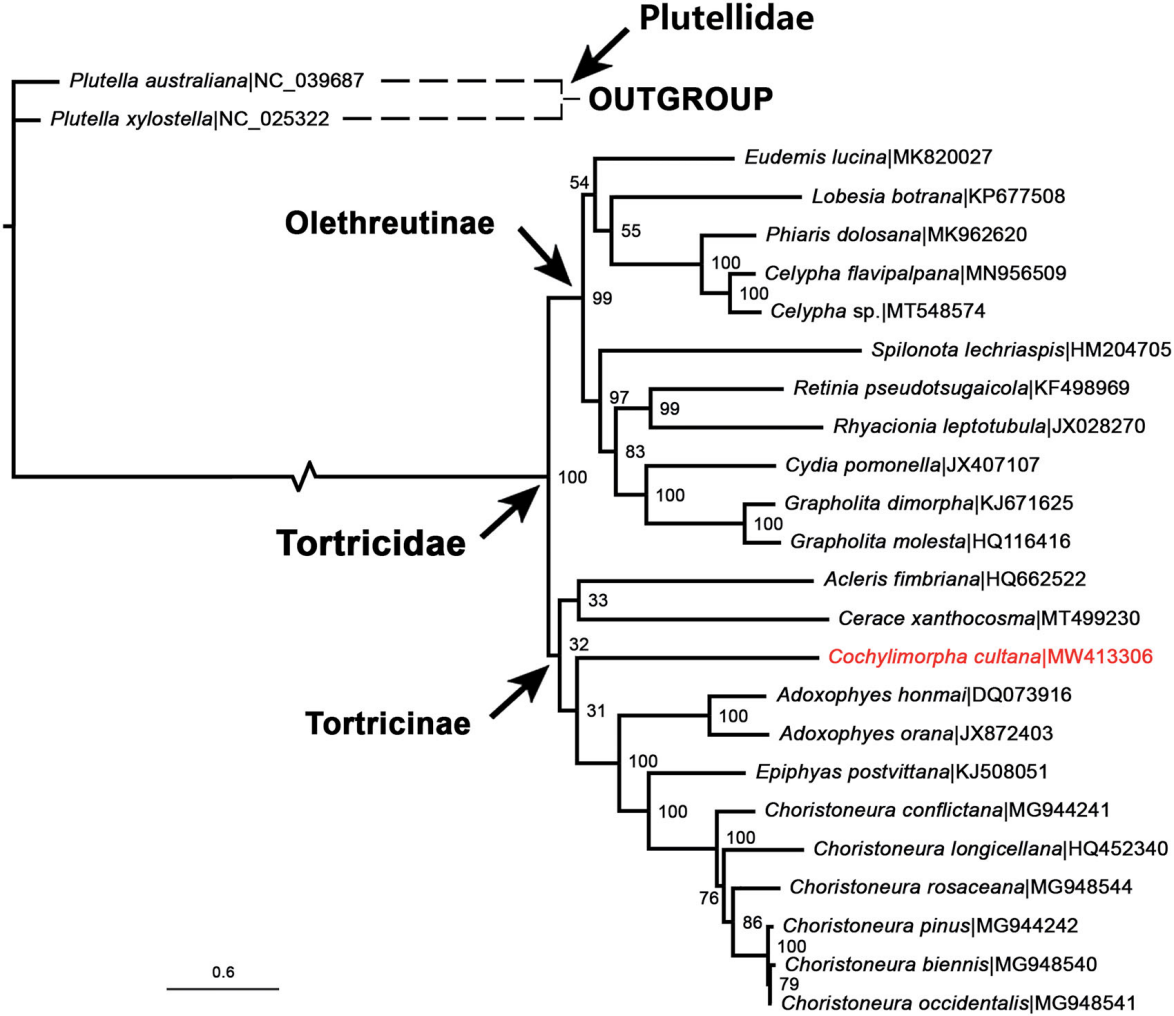
The ML phylogenetic tree was built from *C. cultana* (in red characters) and 24 other species, including two from Plutellidae as outgroup. Bootstrap support values and GenBank accession numbers of the species used were indicated in the tree.

## Data Availability

The data that support the findings of this study are openly available in NCBI at https://www.ncbi.nlm.nih.gov under the accession number MW413306. The associated BioProject, BioSample, and SRA numbers are PRJNA730983, SAMN19238733, and SRR14584945, respectively.
